# Establishment of the Radiologic Tumor Invasion Index Based on Radiomics Splenic Features and Clinical Factors to Predict Serous Invasion of Gastric Cancer

**DOI:** 10.3389/fonc.2021.682456

**Published:** 2021-08-09

**Authors:** Bujian Pan, Weiteng Zhang, Wenjing Chen, Jingwei Zheng, Xinxin Yang, Jing Sun, Xiangwei Sun, Xiaodong Chen, Xian Shen

**Affiliations:** ^1^Department of Gastrointestinal Surgery, The Second Affiliated Hospital, Wenzhou Medical University, Wenzhou, China; ^2^Department of Gastrointestinal Surgery, The First Affiliated Hospital, Wenzhou Medical University, Wenzhou, China

**Keywords:** radiomics, gastric cancer, spleen, radiologic tumor invasion index, serosal invasion

## Abstract

**Background:**

Currently, there are shortcomings in diagnosing gastric cancer with or without serous invasion, making it difficult for patients to receive appropriate treatment. Therefore, we aimed to develop a radiomic nomogram for preoperative identification of serosal invasion.

**Methods:**

We selected 315 patients with gastric cancer, confirmed by pathology, and randomly divided them into two groups: the training group (189 patients) and the verification group (126 patients). We obtained patient splenic imaging data for the training group. A p-value of <0.05 was considered significant for features that were selected for lasso regression. Eight features were chosen to construct a serous invasion prediction model. Patients were divided into high- and low-risk groups according to the radiologic tumor invasion risk score. Subsequently, univariate and multivariate regression analyses were performed with other invasion-related factors to establish a visual combined prediction model.

**Results:**

The diagnostic accuracy of the radiologic tumor invasion score was consistent in the training and verification groups (p<0.001 and p=0.009, respectively). Univariate and multivariate analyses of invasion risk factors revealed that the radiologic tumor invasion index (p=0.002), preoperative hemoglobin <100 (p=0.042), and the platelet and lymphocyte ratio <92.8 (p=0.031) were independent risk factors for serosal invasion in the training cohort. The prediction model based on the three indexes accurately predicted the serosal invasion risk with an area under the curve of 0.884 in the training cohort and 0.837 in the testing cohort.

**Conclusions:**

Radiological tumor invasion index based on splenic imaging combined with other factors accurately predicts serosal invasion of gastric cancer, increases diagnostic precision for the most effective treatment, and is time-efficient.

## Introduction

Gastric cancer (GC) is the fifth most common cancer and is currently the third primary cause of cancer-related deaths worldwide (1). It is important to accurately assess the GC stage preoperatively to determine the most appropriate line of treatment (2). The T4a stage indicates that the tumor has invaded the gastric plasma membrane, suggesting that adjuvant chemotherapy followed by surgery is the most effective plan (3, 4). Patients with a T4 stage and lymph node metastasis are best treated with neoadjuvant chemotherapy (NAC) and may therefore benefit from staging demotion and surgical resection after chemotherapy (5). Therefore, it is necessary to accurately evaluate serosal invasion preoperatively.

Computed tomography (CT) and endoscopic ultrasonography are the most common methods for preoperative staging of GC patients (6). However, these methods have limited sensitivity to detect serosa invasion because the serosa surface may be rough and blurred due to abnormal circumambient adipose tissue, fibrous connective tissue proliferation, or other causes affecting GC staging. Therefore, a novel technique is needed to improve preoperative tumor staging, especially for diagnosing serous invasion (7).

Radiomics, a new diagnostic and predictive method, can easily identify heterogeneity within tissues and uses an automatic high-throughput feature data extraction algorithm to convert image data into data with high-resolution readability (8–10). However, imaging based on tumor region is limited by the accuracy of tumor delineation in patients with early-stage disease and is difficult to use in patients with small tumors or poor gastric dilatation during CT examination. Our previous study have indicated that splenic features and alterations are highly correlated with tumor staging progression and can be used for imaging evaluation (11). In addition, the immune environment may alter the tumor stage and degree of invasion; thus, we intend to predict the tumor stage by evaluating the clinical characteristics of the spleen.

The aim of this study was to use splenic imaging data to explore the correlation between splenic characteristics and serous invasion in GC patients by establishing a prediction model based on the characteristics of splenic imaging. Subsequently, we created a nomogram to help diagnose serous invasion of GC and improve diagnostic accuracy.

## Methods

### Inclusion and Exclusion Criteria

We retrospectively identified patients who underwent GC surgery at The First Affiliated Hospital of Wenzhou Medical University, Wenzhou, China, from January 2015 to May 2017. All patients were subjected to a preoperative CT scan and were pathologically diagnosed with GC by gastroscopy preoperatively and treated with radical gastrectomy; all operations were performed by senior surgeons with experience in performing over 200 radical gastrectomies. Perioperative GC treatment and management were based on the Japanese GC guidelines, 2010 edition. We excluded the following patients: 1) patients who refused to undergo the operation, 2) patients without pre-operative imaging examination or whose imaging data was unavailable, 3) patients with a postoperative diagnosis that differed from those included in our study, and 4) patients with other tumors or other serious organic diseases.

In total, we included 315 patients in this study. The standard of serous invasion was determined using the pathological findings of the surgical specimens as reference standards according to the eighth edition of the American Joint Committee for Cancer and the Union for International Cancer Control Tumor-Node-Metastasis (TNM) classification (12). The Ethics Committee of The First Affiliated Hospital of Wenzhou Medical University approved this research. All patients provided signed informed consent for their clinical data to be collected and analyzed in this study and to be reused in another prospective study (13).

### Radiomics Feature Extraction

All patients underwent an abdominal enhancement CT scan within 30 days of operation. A 64-slice helical CT scanner (Siemens, Germany) of 1.25 mm thickness was used, covering the entire spleen. All CT images were uploaded to ITK-SNAP (version 3.8.0; USA) to protract a semi-automatic splenic mapping area. An experienced general surgeon first drew the spleen, and a radiologist examined the drawing. Medical digital imaging files, in nearly raw raster data format, were used to save the original CT image and region of interest (**Supplementary Figure 1**). Python-based Pyradiomics (version 3.7.2) was used to automate feature extraction. The feature extraction parameters were adjusted as follows: resampledPixelSpacing: [1, 1, 1], normalize: true, normalizeScale: 500, padDistance: 10, Original: (), Wavelet: (), and the rest of the parameters were set to default. A total of 18 first-order features (first order), 14 shape-related features (shape-3D), 22 Gray Level Co-occurrence Matrix1 features, 16 Gray Level Run Length Matrix features, 16 Gray Level Size Zone Matrix features, 5 Neighbouring Gray Tone Difference Matrix features, and 14 Gray Level Dependence Matrix features were extracted, with a total of 833 features after wavelet transform (**Supplementary Material**). Eight hundred and thirty-three radiomic features were extracted from one original and eight wavelet images.

### Screening of Predictive Characteristics and Determining the Radiologic Tumor Invasion Score

To screen spleen-related features and determine the radiologic tumor invasion score, the 315 patients were randomly grouped into either a training cohort or verification cohort in a 3:2 ratio. All 833 features of the 315 patients were z-score normalized by the standard deviation and mean value of the training group. In the univariate logistic analysis with serous invasion, features with a p-value <0.05 were selected for further lasso regression, and a lasso model with minimum lambda in the “cv.lasso” model was chosen to construct a serous invasion risk prediction model (14, 15). Patients in the testing cohort were not involved in feature screening or establishment of the radiologic tumor invasion score and took part in testing the accuracy of the radiologic tumor invasion score.

### Establishment of the Combined Prediction Model

After establishing the radiologic tumor invasion score according to the splenic characteristics, predictive factors were obtained by performing univariate and multivariate analyses between the two cohorts. The results were used to build a risk prediction model and establish the radiologic tumor invasion index, which was applied to the clinic in the form of a visualized nomogram. The stability and reliability of the risk prediction model were evaluated through a C-index calibration curve and decision curve analysis.

### Statistical Analysis

The radiologic tumor invasion index was acquired based on the aforementioned methodology. The distribution uniformity of the continuous parameters was assessed using the Kolmogorov–Smirnov test. The mean ± standard deviation was used to represent the continuous counting data conforming to normal distribution. The continuity variable yielded the optimal cut-off value based on the max Youden index. We used chi-square and Fisher’s precision tests to compare the groups, and continuous variables were analyzed by a rank-sum test. A multivariate logistic regression analysis was performed according to the univariate analysis results to calculate the odds ratios and 95% confidence intervals. An individualized prediction model was set up according to the independent risk factors, and the C-index of the nomogram was reckoned. The SPSS software package (version 22.0; SPSS Inc., Chicago, IL, USA) and R statistics software (version 3.6.1) were used to process all the data and create the risk nomograms. Radiomic features were extracted using the PyRadiomics package (version 3.0.1) in Python (version 3.6.1).

## Results

### Patient Demographics and Clinical Characteristics

All patients were randomly grouped into the training (189 patients) and verification (126 patients) cohorts. The basic clinical features and surgical outcomes revealed no difference between the two cohorts (**Table 1**). Males accounted for 76.9% (146 out of 189) of the training group, and the total median age was 64.9 years. The total incidence of surgical complications was 9.6%, and approximately 21.9% of patients received laparoscopic-assisted surgery in the training group. There was no significant difference between the two groups, indicating that the model was reliable, thereby reducing the error caused by the bias of clinical data and ensuring preciseness of the model data.

**Table 1:** Clinical characteristics of patients.

Random allocation	Training group	Verification group	p
N	189	126	
**BMI(kg/m^2^)**	22.9 ± 3.1	22.7 ± 2.9	0.709
**Age (years)**	64.9 ± 9.3	64.8 ± 9.1	0.360
**Postoperative** **hospital stay (days)**	16 (12-18)	15.5 (11-19)	0.732
**Sex**			0.430
Female	43 (23.1%)	34 (27.0%)	
Male	146 (76.9%)	92 (73.0%)	
**NRS-2002 score**			0.456
1-2	126 (67.0%)	77 (61.1%)	
3-4	50 (26.5%)	42 (33.3%)	
5-6	12 (6.5%)	7 (5.5%)	
**ASA score**			0.778
1-2	155 (81.9%)	105 (83.4%)	
3-4	34 (18.1%)	21 (16.6%)	
**Charlson score**			0.229
0	63 (50.8%)	64 (53.8%)	
1-2	55 (44.4%)	51 (42.9%)	
3-6	6 (4.8%)	4 (3.3%)	
**Hypertension**			0.933
No	137 (72.8%)	92 (73.1%)	
Yes	52 (27.2%)	34 (29.9%)	
**Diabetes**			0.585
No	165 (87.7%)	113 (89.6%)	
Yes	23 (12.3%)	13 (10.4%)	
**Tumor location**			0.777
Cardia	31 (12.6%)	15 (12.6%)	
Corpus	44 (20.5%)	25 (20.5%)	
Antrum	107 (63.4%)	84 (65.4%)	
Diffuse	7 (3.5%)	2 (3.5%)	
**TNM stage**			0.076
1	61 (31.5%)	37 (31.1%)	
2	38 (19.1%)	30 (28.9%)	
3	90 (49.4%)	59 (40%)	
**pT stage**			0.122
1	32 (16.9%)	24 (19.0%)	
2	55 (29.1%)	35 (27.8%)	
3	68 (35.9%)	43 (34.1%)	
4	34 (17.9%)	24 (19.0%)	
**Laparoscopy**			0.054
Yes	147 (78.1%)	86 (68.2%)	
No	41 (21.9%)	40 (31.3%)	
**Total gastrectomy**			0.260
Yes	170 (90.4%)	116 (92.0%)	
NO	19 (9.6%)	10 (8.0%)	
**Complications (greater than grade 2)**			0.185
Yes	170 (90.4%)	116 (92.0%)	
NO	19 (9.6%)	10 (8.0%)	
**Abdominal surgery history**			0.970
Yes	18 (9.6%)	11 (8.5%)	
NO	171 (90.4%)	115 (91.5%)	

Average ± SD or number (%) for results. BMI, body mass index; NRS, Nutritional Risk Screening; ASA, American Society of Anesthesiologists; SD, standard deviation.

### Screening Characteristics and Determining the Radiologic Tumor Invasion Score Based on Splenic Characteristics

**Figure 1:**
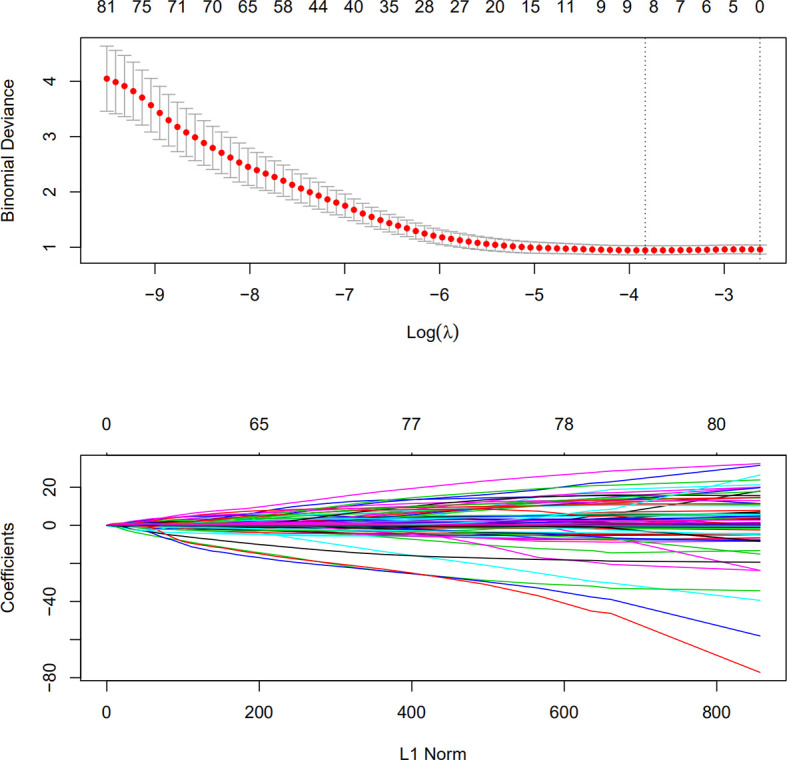
Lasso regression for splenic characteristics in the training group. Characteristics with a logistic regression p-value of <0.05 were used for lasso regression modeling. A lambda (Ln) was selected at −3.833 for the final lasso model.

A detailed list of splenic features for determining the radiologic tumor invasion score is included in this study. In the training group, all 833 features were analyzed by univariate logistic regression analysis, and the features were ranked according to the p-value. A total of 81 features with a p-value <0.05 were selected for further cv.lasso regression (R language glmnet package). The serosal invasion risk model was obtained at a min lambda of −3.833 with eight features (**Figure 1**). The area under the curve (AUC) of the model was 0.76 (p<0.001) in the training group and 0.66 (p=0.009) in the verification group. The radiologic tumor invasion score of all the patients was calculated using the lasso model in R. The risk of serosal invasion increased with the radiologic tumor invasion score, and the cut-off value was set at the max Youden index (−1.566), which yielded the optimal state of specificity and sensitivity. The group with a score of ≤−1.566 was defined as the low-risk group, and the group with a score of >−1.566 was defined as the high-risk group.

### Correlation Analysis Between the Radiomic Tumor Invasion Score and Basic Patient Features

According to the radiologic tumor invasion score, we divided the 189 patients (training cohort) into the high- and low-risk groups. Only TNM and T stage differed significantly between the two groups, while other basic clinical features were not significant (**Table 2**). Moreover, TNM and T stage correlated with the radioactive tumor invasion score (p=0.03 and p=0.019, respectively).

**Table 2:** Clinical data table of radiologic tumor invasion score grouping.

Spleen characteristics groups	Training cohort		
	High-risk group (79)	Low-risk group (110)	p-value
**Age (years)**			0.138
l<70	55 (69.6%)	65 (59.1%)	
≥70	24 (30.4%)	45 (40.9%)	
**Sex**			0.627
Female	17 (21.5%)	27 (24.5%)	
Male	62 (78.4%)	83 (75.5%)	
**NRS-2002 Score**			0.705
1-2	39 (67.9%)	55 (63.2%)	
3-4	32 (67.9%)	41 (29.9%)	
5-6	8 (67.9%)	14 (6.9%)	
**Charlson score**			0.844
0	42 (51.9%)	58 (51.3%)	
1-2	35 (45.7%)	48 (44.9%)	
3-6	2 (2.4%)	4 (3.8%)	
**Hypertension**			0.261
Yes	25 (32.1%)	34 (25.6%)	
No	54 (67.9%)	76 (74.4%)	
**Diabetes**			0.610
Yes	7 (9.9%)	11 (12.0%)	
No	72 (90.1%)	99 (88.0%)	
**Tumor location**			0.321
Cardia	9 (12.3%)	13 (9.0%)	
Corpus	20 (25.9%)	33 (28.2%)	
Antrum	45 (55.5%)	57 (56.4%)	
Diffuse	5 (6.3%)	7 (6.4%)	
**pN stage**			0.230
N0	30 (38.3%)	47 (41.9%)	
N+	49 (61.7%)	63 (58.1%)	
**pT stage**			0.019*
1-2	25 (31.6%)	53 (48.2%)	
3	22 (27.8%)	32 (29.1%)	
4	32 (40.5%)	25 (22.7%)	
**pTNM stage**			0.030*
1	23 (29.1%)	49 (44.5%)	
2	18 (22.8%)	28 (25.5%)	
3	38 (48.1%)	33 (30%)	
**Differentiation**			0.732
Yes	61 (77.2%)	87 (79.1%)	
No	18 (22.8%)	23 (20.9%)	

Average SD or number (%) for results. *Showing statistical significance (P < 0.05). Calculation of variables whose theoretical number was <10 by Fisher accurate probability test. BMI, body mass index; NRS, Nutritional Risk Screening; TNM, Tumor Node Metastasis; SD, standard deviation.

### Univariate and Multivariate Analyses of Risk Factors for Serosal Invasion in the Training Cohort

The univariate analysis indicated that the radiologic tumor invasion score (p<0.001), ratio between neutrophil and lymphocyte (p=0.043), ratio between platelet and lymphocyte (PLR) (p=0.023), and preoperative hemoglobin (p=0.029) were relevant to serosal invasion (**Table 3**). The multivariate logistic regression analysis revealed that the radiologic tumor invasion score (p=0.002), PLR (p=0.031), and preoperative hemoglobin (p=0.042) were independent risk factors for predicting serosal invasion (**Table 4**). The probability of serosal invasion was higher in the high-risk group than in the low-risk group (hazard ratio=3.562).

**Table 3:** Univariate analysis table of serosal invasion.

Factors	Training cohort		Univariate analysis
	statistics	HR (95%CI)	p-value
**Splenic characteristics group**		6.231	<0.001***
High-risk	79 (41.7%)		
Low- risk	110 (58.2%)		
**Age (years)**		1.279	0.376
<70	120 (64.1%)		
≥70	69 (35.9%)		
**Sex**		1.021	0.952
Female	44 (24.4%)		
Male	145 (75.6%)		
**NRS-2002 score**		0.863	0.863
1-3	125 (32.4%)		
4-6	64 (67.6%)		
**Hypertension**		1.382	0.303
Yes	59 (27.3%)		
No	130 (72.7%)		
**Diabetes**		0.865	0.221
Yes	18 (6.3%)		
No	171 (6.3%)		
**Differentiation**		0.883	0.443
Differentiation	148 (74.6%)		
Non differentiation	41(7.9%)		
**NLR**		2.132	0.043*
≥2.75	60 (28.3%)		
<2.75	129(71.7%)		
**PLR**		3.834	0.023*
≥92.8	67 (40.0%)		
<92.8	122(60.0%)		
**Preoperative_hemoglobin (g)**		3.903	0.029*
≥100	147 (75,2%)		
<100	42 (24.8%)		

*Showing statistical significance (p < 0.05). HR, hazard ratio; CI, confidence interval; BMI, body mass index; PLR, ratio between platelet and lymphocyte; Nutritional Risk Screening; ASA, American Society of Anaesthesiologists; NLR, ratio between neutrophil and lymphocyte. ***p < 0.001.

**Table 4:** Multivariate analysis table of serosal invasion.

Factors	Training cohort		Multifactor analysis
	Statistics	HR (95%CI)	p-value
**Splenic characteristics group**		3.562	0.002**
High-risk	79 (41.7%)		
Low- risk	110 (58.2%)		
**NLR**		1.412	0.142
≥2.75	60 (28.3%)		
<2.75	129 (71.7%)		
**PLR**		2.439	0.031*
≥92.8	67 (40.0%)		
<92.8	122 (60.0%)		
**Preoperative_hemoglobin (g)**		2.103	0.042*
≥100	147 (75,2%)		
<100	42 (24.8%)		

*Showing statistical significance (p < 0.05). HR, hazard ratio; CI, confidence interval;

PLR, ratio between platelet and lymphocyte; NLR, ratio between neutrophil and lymphocyte. **p < 0.01.

### Establishment and Validation of the Prediction Model

**Figure 2:**
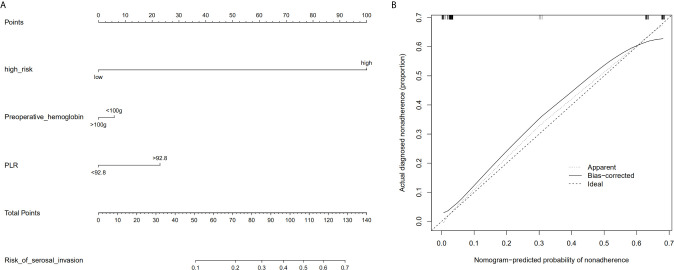
Radiomic nomogram based on radiologic tumor invasion score and clinical factors and a calibration curve of the radiomics nomogram in the training cohort. **(A)** The nomogram consists of three indicators, namely, radiation tumor invasion score, preoperative hemoglobin, and platelet and lymphocyte ratio. By adding the three scores, the nomogram can quickly calculate the probability of serosal invasion. **(B)** The calibration curve depicts the consistency between the predicted values of serosal invasion and the actual observed values. The y-axis represents the actual value. The x-axis represents the predicted value. The more consistent the dotted and solid lines are, the better the predictive power of the model is. PLR, ratio between platelet and lymphocyte.

Based on the radiologic tumor invasion score, PLR, and preoperative hemoglobin, we developed a prediction model and established the radiologic tumor invasion index for serosal invasion (**Figure 2**) in the training cohort. The final visual prediction model calculated the serous invasion rate of each patient based on the score of each index. The calibration curve of the radiomic nomogram demonstrated consistency between the predictive risk and the observed probability, which was validated in the testing cohort (**Figure 4**). The Hosmer–Lemeshow test was not significant (p=0.466), suggesting that there was no apparent departure.

### Clinical Benefits

**Figure 3:**
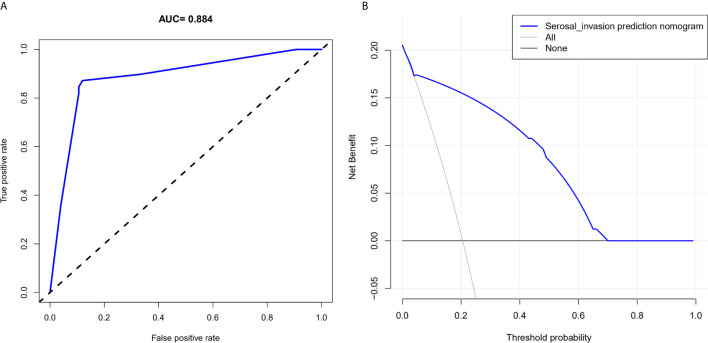
Area under the curve of the prediction model and decision curve analysis for the radiomics nomogram in the training cohort. **(A)** The area under the curve represents the reliability of the model; the larger the value, the more reliable the model. **(B)** The y-axis measures net income. The gray line indicates that the patient has serous invasion, and the black line indicates that the patient has no serous invasion. The blue line represents the benefit of the patient after using the prediction model.

**Figure 4:**
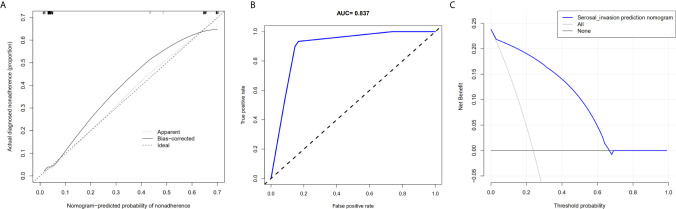
Calibration curve of the radiomics nomogram, area under the curve of the prediction model, and decision curve analysis for the radiomics nomogram in the testing cohort. **(A)** The calibration curve depicts the consistency between the predicted values of serosal invasion and the actual observed values. **(B)** The area under the curve represents the reliability of the model. **(C)** The blue line represents the benefit of the patient after using the prediction model.

The AUC in the training and testing cohorts is 0.884, which is high and indicates that the model is reliable, and 0.837, respectively (**Figure 4**). The decision curve illustrated that if the actual incidence of serosal invasion was ≥10%, predicting serosal invasion using radiomics nomogram led to more benefits than the treatment-all or treatment-none scheme both in the training and testing cohorts (**Figures 3** and **4**). Thus, the net benefit can be compared and obtained using radiomics nomogram.

## Discussion

Accurate preoperative staging is important to ensure that patients receive appropriate treatment (3, 16). The Chinese Society of Clinical Oncology guidelines recommend preoperative NAC treatment for GC stage T4a or higher but do not recommend the same for T3 (17, 18). Endoscopic ultrasound is approximately 65% accurate in differentiating T3 tumors. CT scans are reported to overestimate T3 pathology and underestimate T4 pathology, with an overall accuracy rate of approximately 77%. To avoid unnecessary NAC, it is important to accurately distinguish T3 from T4a patients (19, 20).

The distinguishing parameter between T3 and T4a is serous membrane invasion; therefore, it is important to establish a method that can accurately and specifically detect serous invasion (21–23). The spleen is closely related to tumor development, and tumor development can be predicted by imaging splenic features (24, 25). Moreover, radiomics has the ability to quantitatively identify tissue characteristics, which often have a causal relationship with biological prognosis or genes, allowing it to establish the relationship between textural and biological features (26–28).

Recently, the spleen has been reported to promote tumor progression and metastasis in many types of tumors. Gay et al. demonstrated that non-leukocyte populations, such as platelets, outside the tumor micro-circles promote tumor metastasis (29). Furthermore, Han et al. demonstrated that the mouse model of hepatocellular carcinoma was successfully established, and the splenic erythrocyte-like cells (Ter cells) promote tumor progression (30). Thus, the spleen is closely related to the tumor and can be used to predict tumor development. Therefore, we screened some splenic features associated with serosal invasion to establish an evaluation index called the radiologic tumor invasion score. This score is minimally affected by tumor size and location and is suitable for most GC patients. Moreover, the score can predict serosal invasion and was validated by our verification group.

We divided the patients into a high- and low-risk group using the score of reflex tumor invasion and analyzed their basic characteristics. We found that only TNM and T stages differed significantly between the two groups (p=0.030 and p=0.019), with the high-risk group often having late TNM and T-stage disease. A study have revealed that the spleen of cancer patients is usually larger than that of normal people, especially in patients with advanced cancer, suggesting that the size of the spleen is closely associated with tumor progression (31). Similarly, our risk analysis of multivariate invasion demonstrated that there was a high risk of serous invasion in the high-risk group (hazard ratio=3.562, p=0.002).

Overall, we established and validated a prediction model based on the radiologic tumor invasion score for preoperatively predicting serosal invasion in GC patients. The radiologic tumor invasion score can successfully distinguish the risk of serosal invasion between the two groups. The serosal invasion prediction model was established based on three independent risk factors obtained from multivariate analysis. The visual prediction model can thus calculate the serous invasion rate of each patient based on the score of each index in the chart (32). The calibration curve of the nomogram revealed that the predictive risk and observed probability were in agreement, and the Hosmer–Lemeshow test revealed that there was no obvious departure. For clinical use, the decision curve demonstrated that if the threshold probability of serosal invasion is ≥10%, predicting serosal invasion using radiomics nomogram led to more benefits than the treatment-all or treatment-none regimen (33, 34). More importantly, this model has a discrimination concordance index of 0.884, and the parameters in this model are readily available, making it easy to accurately assess serosal invasion. To the best of our knowledge, this is the first study to reveal an intrinsic link between splenic imaging features and tumor serous invasion.

This study has several limitations. First, only confirmed GC patients were included in our study, which may overestimate the ability of the radiologic tumor invasion score to stage GC. Second, the sample size was small; thus, larger sample sizes are needed to validate our results. Third, the lack of advanced dimensionality reduction technologies may have led to some bias. Finally, we built the model using only machine learning algorithms; advanced deep learning technology should be used in future studies.

In conclusion, this study demonstrates that the spleen imaging-based radiological tumor invasion score combined with preoperative hemoglobin and PLR can distinguish between T3 and T4 GC patients, making it easier for patients to receive NAC treatment.

## Data Availability Statement

The raw data supporting the conclusions of this article will be made available by the authors, without undue reservation.

## Ethics Statement

The studies involving human participants were reviewed and approved by The Ethics Committee of The First Affiliated Hospital of Wenzhou Medical University. The patients/participants provided their written informed consent to participate in this study. Written informed consent was obtained from the individual(s) for the publication of any potentially identifiable images or data included in this article.

## Author Contributions

BP: performed the experiments, analyzed and interpreted the data, and drafted the manuscript. Performed the experiments and statistical analysis. WZ, WC, JZ: acquired the data and material support. XY, JS, XWS, XC: analyzed and interpreted the data, revised the manuscript, and finally approved the version of the manuscript for publication. XS: made contribution to the conception and design,analyzed and interpreted the data, supervised the study, provided the project funding, revised the manuscript and finally approved the version of the manuscript for publication. All authors contributed to the article and approved the submitted version.

## Funding

This study was supported by the General Scientific Research Project of the Education Department of Zhejiang Province (No. Y201941489), a project of the regional diagnosis and treatment center of the Health Planning Committee (No. JBZX-201903).

## Conflict of Interest

The authors declare that the research was conducted in the absence of any commercial or financial relationships that could be construed as a potential conflict of interest.

## Publisher’s Note

All claims expressed in this article are solely those of the authors and do not necessarily represent those of their affiliated organizations, or those of the publisher, the editors and the reviewers. Any product that may be evaluated in this article, or claim that may be made by its manufacturer, is not guaranteed or endorsed by the publisher.
