# Supplementary material for: Establishment of the Radiologic Tumor Invasion Index Based on Radiomics Splenic Features and Clinical Factors to Predict Serous Invasion of Gastric Cancer
Source: Front Oncol. 2021 Aug 9;11:682456. doi: 10.3389/fonc.2021.682456 (PMC8381151; doi:10.3389/fonc.2021.682456)
Supplement: Supplementary file 2 [file DataSheet_2.docx]

Supplementary Material

# Supplementary Figures


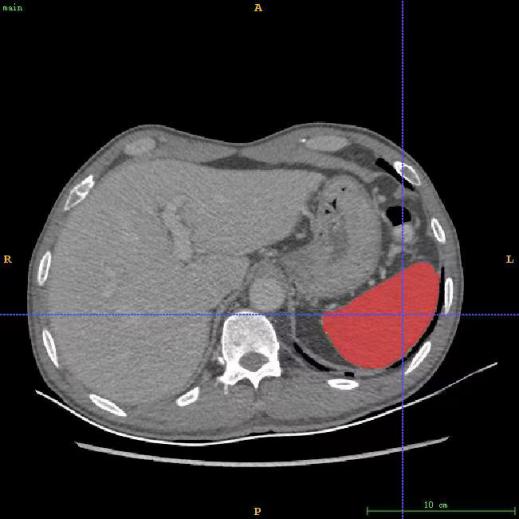

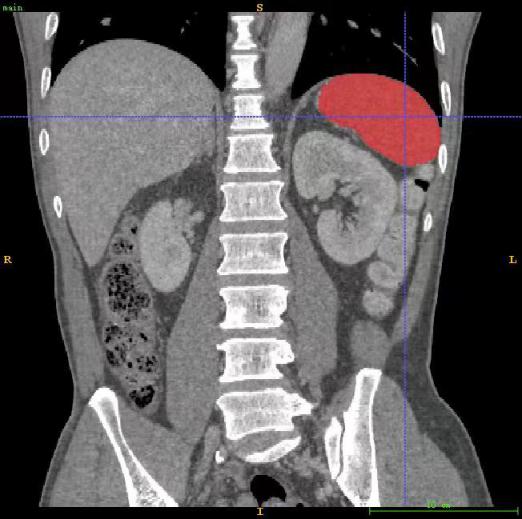


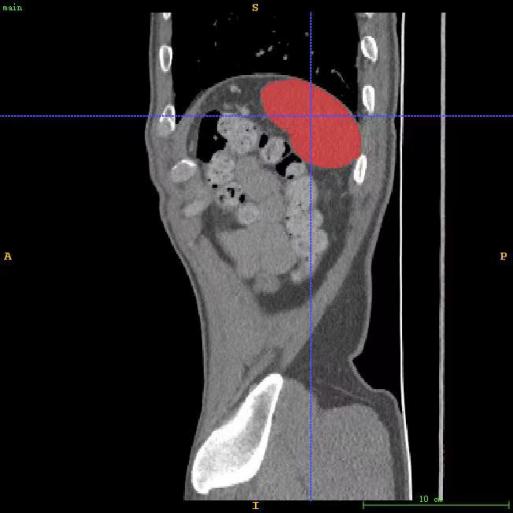

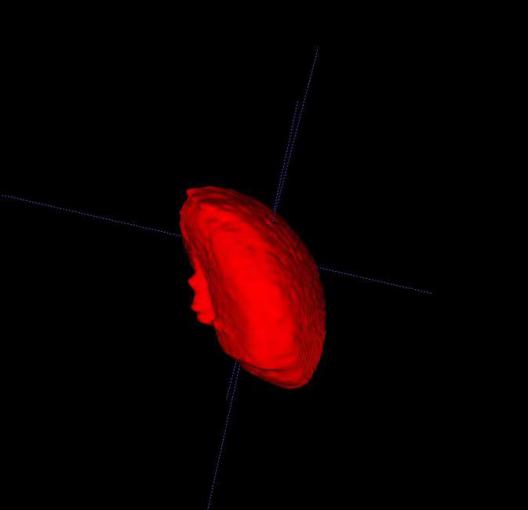


**Supplementary Figure 1.** Computed tomography images. After marking the splenic region in the ITK-SNAP software, asemi-automatic drawing is adopted for the splenic region, followed by selection and three-dimensional reconstruction.

# Radiomic Feature Extraction Parameters

（1）.First-order statistics(18features)：

original_firstorder_10Percentile，original_firstorder_90Percentile，original_firstorder_Energy，original_firstorder_Entropy，original_firstorder_InterquartileRange，original_firstorder_Kurtosis，original_firstorder_Maximum，original_firstorder_MeanAbsoluteDeviation，original_firstorder_Mean,original_firstorder_Median,original_firstorder_Minimum,original_firstorder_Range,original_firstorder_RobustMeanAbsoluteDeviation,original_firstorder_RootMeanSquared，original_firstorder_Skewness，original_firstorder_TotalEnergy，original_firstorder_Uniformity，original_firstorder_Variance.

（2）.Shape Features(3D)(14features): original_shape_Elongation,original_shape_Flatness,original_shape_LeastAxisLength,original_shape_MajorAxisLength,original_shape_Maximum2DDiameterColumn,original_shape_Maximum2DDiameterRow,original_shape_Maximum2DDiameterSlice,original_shape_Maximum3DDiameter,original_shape_MeshVolume,original_shape_MinorAxisLength,original_shape_Sphericity,original_shape_SurfaceArea,original_shape_SurfaceVolumeRatio,original_shape_VoxelVolume.

（3）. Gray Level Co-occurrence Matrix (GLCM) Features(22features): original_glcm_Autocorrelation,original_glcm_JointAverage,original_glcm_ClusterProminence,original_glcm_ClusterShade,original_glcm_ClusterTendency,original_glcm_Contrast,original_glcm_Correlation,original_glcm_DifferenceAverage,original_glcm_DifferenceEntropy,original_glcm_DifferenceVariance,original_glcm_JointEnergy,original_glcm_JointEntropy,original_glcm_Imc1,original_glcm_Imc2,original_glcm_Idm,original_glcm_Idmn,original_glcm_Id,original_glcm_Idn,original_glcm_InverseVariance,original_glcm_MaximumProbability,original_glcm_SumEntropy,original_glcm_SumSquares.

（4）.Gray Level Size Zone Matrix (GLSZM) Features(16features): original_glszm_GrayLevelNonUniformity,original_glszm_GrayLevelNonUniformityNormalized,original_glszm_GrayLevelVariance,original_glszm_HighGrayLevelZoneEmphasis,original_glszm_LargeAreaEmphasis,original_glszm_LargeAreaHighGrayLevelEmphasis,original_glszm_LargeAreaLowGrayLevelEmphasis,original_glszm_LowGrayLevelZoneEmphasis,original_glszm_SizeZoneNonUniformity,original_glszm_SizeZoneNonUniformityNormalized,original_glszm_SmallAreaEmphasis,original_glszm_SmallAreaHighGrayLevelEmphasis,original_glszm_SmallAreaLowGrayLevelEmphasis,original_glszm_ZoneEntropy,original_glszm_ZonePercentage,original_glszm_ZoneVariance.

（5）.Gray LevelRun Length Matrix (GLRLM) Features(16features): original_glrlm_GrayLevelNonUniformity,original_glrlm_GrayLevelNonUniformityNormalized,original_glrlm_GrayLevelVariance,original_glrlm_HighGrayLevelRunEmphasis,original_glrlm_LongRunEmphasis,original_glrlm_LongRunHighGrayLevelEmphasis,original_glrlm_LongRunLowGrayLevelEmphasis,original_glrlm_LowGrayLevelRunEmphasis,original_glrlm_RunEntropy,original_glrlm_RunLengthNonUniformity,original_glrlm_RunLengthNonUniformityNormalized,original_glrlm_RunPercentage,original_glrlm_RunVariance,original_glrlm_ShortRunEmphasis,original_glrlm_ShortRunHighGrayLevelEmphasis,original_glrlm_ShortRunLowGrayLevelEmphasis.

（6）.Neighbouring Gray Tone Difference Matrix (NGTDM) Features(5features): original_ngtdm_Busyness,original_ngtdm_Coarseness,original_ngtdm_Complexity,original_ngtdm_Contrast,original_ngtdm_Strength.

1. .Gray Level Difference Matrix (GLDM) Features(14features)：original_gldm_DependenceEntropy，original_gldm_DependenceNonUniformity，original_gldm_DependenceNonUniformityNormalized，original_gldm_DependenceVariance，original_gldm_GrayLevelNonUniformity，original_gldm_GrayLevelVariance，original_gldm_HighGrayLevelEmphasis，original_gldm_LargeDependenceEmphasis，original_gldm_LargeDependenceHighGrayLevelEmphasis，original_gldm_LargeDependenceLowGrayLevelEmphasis，original_gldm_LowGrayLevelEmphasis，original_gldm_SmallDependenceEmphasis，original_gldm_SmallDependenceHighGrayLevelEmphasis，original_gldm_SmallDependenceLowGrayLevelEmphasis
2. .8-Fold Radiomic features (except Shape Features) from 8 wavelet images(728 features in total).

1. **Supplementary Figures**

**Figure s1. linear SVM model in valid cohort**


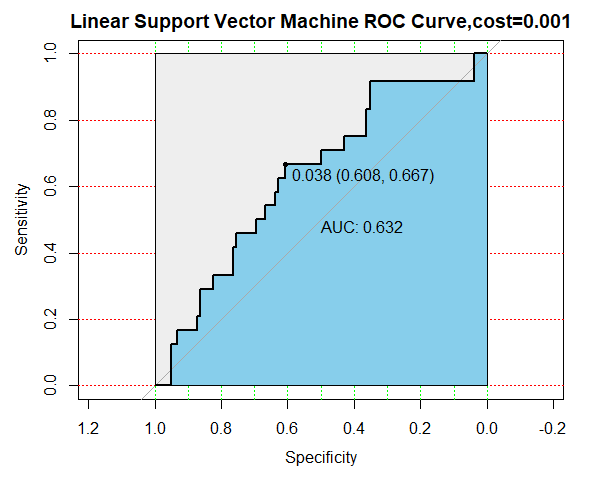


**Figure s2. Random forest model in valid cohort**


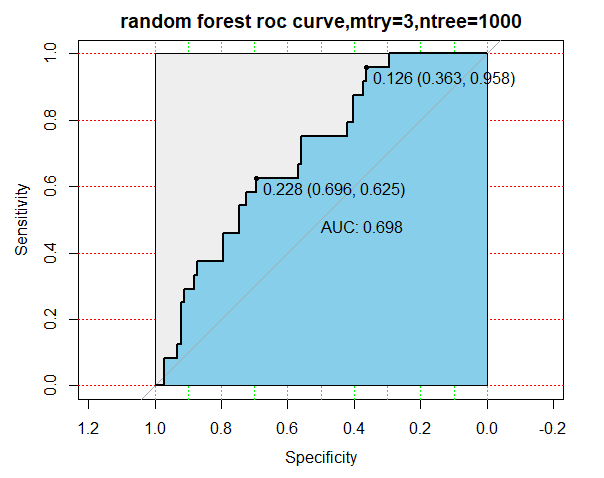


**Figure s3. LASSO model in valid cohort**

**
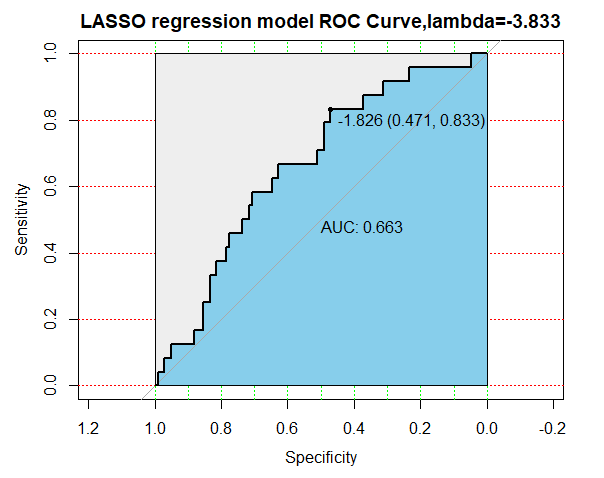
**

1. **Result s1. Lasso regression model formula**

Lasso regression model:

[radiomic score = -1.624147865+original_glrlm_RunVariance*0.142532106+

original_glszm_LargeAreaLowGrayLevelEmphasis*0.143264851+

wavelet.LHL_glszm_SmallAreaEmphasis*0.169675709+

wavelet.LHH_firstorder_Median*-0.157857841+

wavelet.HHL_firstorder_Kurtosis*0.316897782+

wavelet.HHH_glrlm_RunEntropy*-0.302448733+

wavelet.HHH_glrlm_RunLengthNonUniformityNormalized*0.017168423+

wavelet.LLL_glszm_LargeAreaLowGrayLevelEmphasis*0.100437653]

**
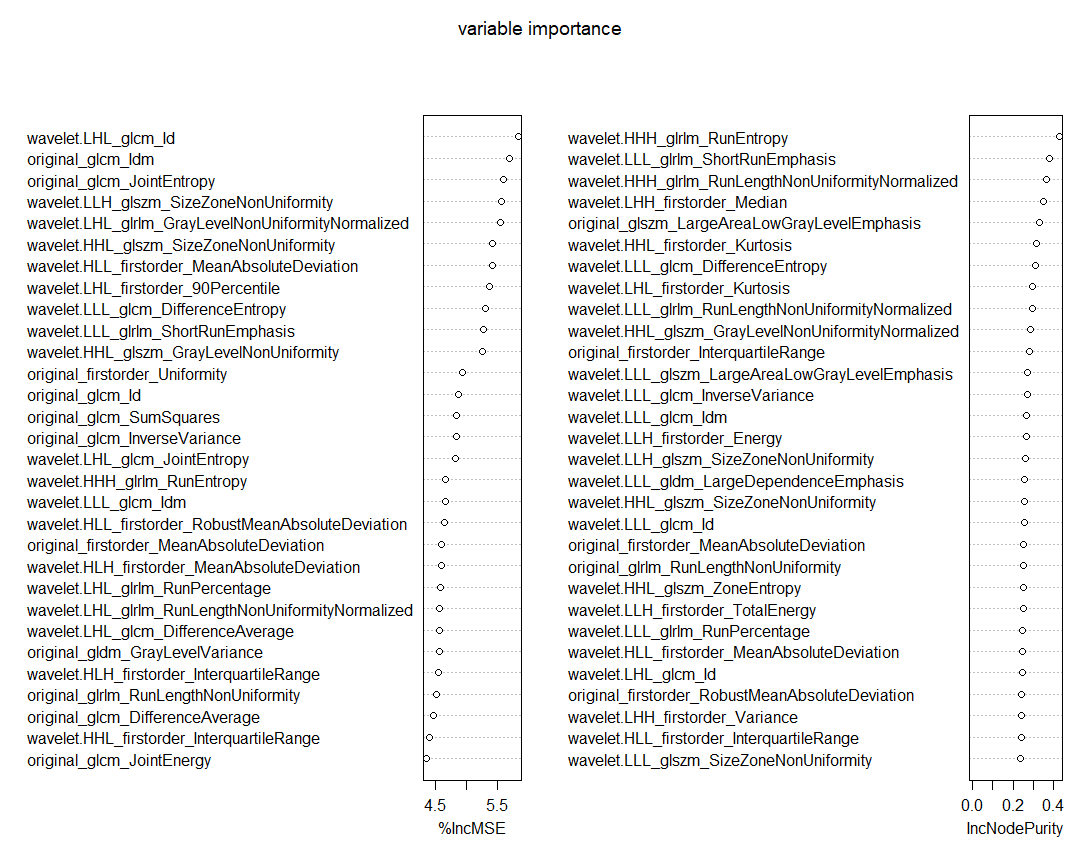
**
